# Comparison of Bioelectrical Impedance Analysis with DXA in Adolescents with Cystic Fibrosis before and after a Resistance Training Intervention

**DOI:** 10.3390/ijerph19074037

**Published:** 2022-03-29

**Authors:** Clifton J. Holmes, Susan B. Racette, Leslie Symonds, Ana Maria Arbeláez, Chao Cao, Andrea Granados

**Affiliations:** 1 Program in Physical Therapy, Washington University School of Medicine, St. Louis, MO 63108, USA; racettes@wustl.edu (S.B.R.); caochao@wustl.edu (C.C.); 2 Center for Human Nutrition, Department of Medicine, Washington University School of Medicine, St. Louis, MO 63108, USA; 3 Department of Pediatrics, Washington University School of Medicine, St. Louis, MO 63108, USA; lsymonds@wustl.edu (L.S.); aarbelaez@wustl.edu (A.M.A.); 4 Nicklaus Children’s Hospital, Division of Pediatric Endocrinology and Metabolism, Miami, FL 33155, USA; andrea.granados@nicklaushealth.org

**Keywords:** body composition, fat mass, muscle mass, nutritional status, diabetes, impaired glucose tolerance

## Abstract

Background: The purpose of this pilot study was to compare body composition metrics obtained by two portable bioelectrical impedance analysis (BIA) devices with dual-energy X-ray absorptiometry (DXA) among adolescents with cystic fibrosis (CF) before and after a resistance exercise training program. Methods: Participants with CF were assessed using DXA, single-frequency BIA (SFBIA), and multiple-frequency BIA (MFBIA) to quantify percent body fat (%Fat), fat mass (FM), and fat-free mass (FFM) at baseline and after a home-based resistance training intervention comprised of 36, 1 h sessions completed in 12–14 weeks. Repeated measures analysis of variance, paired samples *t*-tests, Cohen’s d effect sizes, and Pearson’s correlations were used to compare differences between and within methods at baseline and post-intervention. Results: Ten participants (15.8 ± 2.2 yr, 60.1 ± 15.1 kg) completed the assessments. At baseline, both SFBIA and MFBIA scales significantly underestimated %Fat and FM and overestimated FFM, with small to moderate effect sizes. Post-intervention, small, non-significant differences were found between DXA and both BIA scales for all body composition metrics. Significant changes in %Fat and FFM were observed with DXA. MFBIA displayed less constant error than SFBIA when compared to DXA for pre- and post-intervention assessments for %Fat (MFBIA: pre and post −2.8 and −0.8 vs. SFBIA: −4.6 and −2.0), FM (−0.4 and −0.4 vs. −3.0 and −1.1), and FFM (+0.8 and +0.6 vs. +3.1 and +1.3). Near-perfect correlations were observed at both time points between DXA and each BIA scale. *Conclusions:* Portable BIA results should be interpreted with caution, and further validation studies in CF patients are needed prior to clinical use.

## 1. Introduction

Cystic fibrosis (CF) is a multisystem disease caused by mutations in the cystic fibrosis transmembrane conductance regulator gene, which results in shortened lifespan due to pulmonary failure and multisystem complications [1]. CF-related diabetes (CFRD) has emerged as the most common extrapulmonary complication in this population, contributing to morbidity through poor nutritional status. Because CFRD can begin developing several years before the diagnosis of diabetes, and the risk increases with age, it is recommended that testing for CFRD begin as early as age 10 years [2,3,4,5,6]. Exercise training has been recommended as a complementary therapeutic method to improve health-related quality of life, physical work capacity, muscle strength, and respiratory function for patients with CF [7,8]. In particular, chronic resistance training is suggested as a potentially promising modality for enhancing pulmonary function, inspiratory muscle strength, exercise tolerance, glucose tolerance, and body composition [8,9,10,11,12]. Excess adiposity contributes to abnormal glycemic control, whereas lean body mass was associated inversely with insulin resistance and the risk of prediabetes in a large cohort representative of the general population. Moreover, increases in lean body mass have been associated with protection against insulin resistance and prediabetes [13,14,15]. Because body composition is considered an important part of the nutritional status among individuals with CF, assessing body composition changes over time may become an important mechanism to guide treatment decisions and health outcomes in the CF population.

A common metric used by physicians, dietitians, and other clinicians to assess nutritional status is body mass index (BMI), as there are evidence-based reference values for all ages across many populations. Focusing exclusively on BMI, however, may miss important information about body composition, which is a key determinant of health outcomes in children and adults with and without CF [16,17,18]. When assessing body composition, dual-energy X-ray absorptiometry (DXA) is the gold standard method for evaluating bone mass and is the most frequently used method for assessing percent body fat (%Fat), fat mass (FM), and fat-free mass (FFM) in both clinical and research settings [19]. DXA is preferred primarily due to it being a 3-compartment or multi-compartment model that quantifies FM, bone mineral content, and fat-free, non-bone lean mass, which increases accuracy relative to 2-compartment models (e.g., hydrostatic weighing) and reduces assumptions on which body composition estimates are based [20,21,22,23]. DXA scans are recommended to assess bone mineral density among patients with CF, but body composition assessment is not the standard of care to assess body composition. Furthermore, DXA scanners are expensive, require technical expertise to operate, and are not available in many clinical centers. Scheduling additional visits with patients for the purpose of body composition assessment may not be feasible due to long travel distances to their clinical care centers or to a facility with a DXA scanner [24]. An additional concern with DXA is the radiation exposure which, although low-dose, similar to a standard X-ray, and generally considered safe, may pose future health risks with repeated doses [25,26]. Therefore, performing frequent DXA measurements longitudinally outside of research settings is extremely difficult.

BIA devices are practical, safe, and inexpensive tools to analyze body composition in research, clinical, and home-based settings. Importantly, a high level of technical expertise is not required to operate the majority of the current scales available to the general public. With the growing number of available portable BIA scales, previous studies have demonstrated the validity of both single-frequency BIA (SFBIA) and multi-frequency BIA (MFBIA) devices, concluding that BIA may be used as an alternative to DXA for body composition assessment [27,28]. Although commercially available BIA devices offer a viable alternative to traditional body composition methods, accuracy and reliability vary widely among BIA instruments. SFBIA demonstrates the largest differences when compared to DXA, with the inaccuracy increasing in conjunction with higher levels of BMI in various populations [29,30]. MFBIA devices are thought to have higher accuracy because different parts of the body having varying levels of resistance require specific current frequencies to detect the distribution of extracellular and intracellular water. With SFBIA utilizing only one frequency, it does not allow for current penetration of all cell membranes and the most accurate assessment of total body water [31,32]. The accuracy of portable BIA scales in people with CF is inconsistent in the scientific literature [33,34,35]. Moreover, investigations involving adolescents with CF are lacking. The aim of this pilot study was to compare %Fat, FM, and FFM obtained by two portable, commercial BIA scales and DXA among adolescents with CF and impaired glucose tolerance before and after a resistance training program. We hypothesized that the MFBIA would demonstrate greater accuracy and agreement with criterion DXA-derived body composition metrics than SFBIA. 

## 2. Materials and Methods

### 2.1. Participants and Study Design

The current pilot study and outcome measures were secondary, derived from a larger study investigating the feasibility and effects of home-based resistance exercise training in adolescents with CF and impaired glucose tolerance [36]. Adolescents aged 10 to 18 years with CF and pancreatic insufficiency were invited to participate in this prospective study. Additional eligibility criteria were that participants had to be clinically stable, without evidence of deterioration from previous pulmonary function tests, and no hospitalizations or steroid use for 1 month prior to the study visit. Participants were included in the study if they had a history of CFRD without fasting hyperglycemia and were not on insulin therapy. Eligible diagnoses included impaired fasting glucose (≥100–125 mg/dL), impaired glucose tolerance (2 h glucose 140–199 mg/dL), or indeterminate hyperglycemia (1 h glucose ≥200 mg/dL and normal fasting and 2 h glucose. Additional exclusion criteria were use of medications affecting glucose homeostasis, pulmonary exacerbations or admissions in the 4 weeks prior to the study visit, and pregnancy. Participants were required to be motivated to exercise during the study period and to have a smartphone, tablet, or computer available at home. All participants and their parents or legal guardians signed informed consent or provided verbal assent (if < 18y). This study was approved by the Washington University in St. Louis Institutional Review Board (IRB#201806163).

Participants attended the Washington University CF Center for clinical visits and the Washington University Clinical Translation Research Unit for research study visits. Participants completed two study assessment visits, one at baseline and the second after a home-based resistance exercise training intervention. Participants fasted for a minimum of 8 h (ad libitum water intake was allowed) before both study visits and were instructed to refrain from exercising for at least 24 h prior to testing. 

### 2.2. Anthropometric Measurements

Weight was measured on a digital scale (SECA model 6841321107) with participants wearing light clothing. Standing height without shoes was measured using a wall-mounted stadiometer (SECA model 240) and recorded to the nearest 0.1 cm). For descriptive purposes, BMI was calculated as weight (kg) ÷ height (m)^2^. 

### 2.3. Body Composition Assessments

#### 2.3.1. Bioelectrical Impedance Analysis

Two commercially available segmental, hand-to-foot BIA scales were used: SFBIA (BC-558 Ironman; Tanita Corp., Tokyo, Japan) and MFBIA (BC-1500 Plus Ironman; Tanita Corp., Tokyo, Japan). The SFBIA device operates at 50 kHz, while the MFBIA device operates at 6.25 and 50 kHz. Both devices contain eight contact electrodes, two pairs of electrodes being coupled to a metal platform for the feet and two pairs for hand grasping. The measurement of body composition was performed by a trained investigator using manufacturer’s instructions and recommendations from previous research [37,38,39]. Prior to each measurement, participants voided, and hydration status was assessed using urine-specific gravity testing. All participants produced specific gravity values of <1.030, indicating adequate hydration. Participants were instructed to wipe both hands and feet with a damp cloth towel to ensure that skin was free of excessive cream, oil, or other surface materials that could interfere with measurements. Next, participants were asked to stand in a stable position prior to stepping onto the scale, with their toes and heels in contact with the anterior and posterior electrodes of the weighing platform. The electrode-containing grips were then grasped by the participants, and arms were abducted approximately 30^°^ from the trunk. The BIA scales were set to use the ‘normal’ (nonathletic) proprietary algorithm. Body mass (kg) and whole-body %Fat values were obtained from the scales directly; FM and FFM values were calculated using the following equations:

FM (kg) = body mass (kg) × (%Fat/100)

FFM (kg) = body mass (kg) − FM (kg)

#### 2.3.2. Dual Energy X-ray Absorptiometry

Criterion whole-body measures of FM and %Fat were obtained using a Lunar Prodigy Advance DXA scanner (General Electric Healthcare, Encore Software Version 16). Prior to each scan, the DXA scanner was calibrated according to the manufacturer’s instructions using a standard calibration block. Whole-body scans were performed with participants in a supine position on the scanning bed with hands at their sides. FFM was calculated using the equation outlined in the previous section. 

### 2.4. Resistance Exercise Training Intervention

After baseline testing, participants began a home-based resistance training program consisting of 36 whole-body exercise sessions performed three times per week on nonconsecutive days, allowing for at least 24 h of recovery between sessions. The program was designed to be completed in 12 weeks, but an additional 2 weeks were allowed to account for missed sessions due to unforeseen situations (e.g., illness or travel). The participants were given a set of weight-adjustable dumbbells and assigned a personal trainer to supervise their at-home exercise sessions via live video calls. Emphasis was placed on volume progression instead of load progression, where set number was increased at weeks 5 and 10 unless individual performance dictated otherwise, in which case adjustments were made to best suit the participant. The set number progressed from 1–4 sets, with a repetition range of 8–15. Relative load (%1RM) remained constant at ~60% 1RM. In order to maintain a load of 60%, weight was increased from subsequent sessions if the subject was able to complete ≥3 repetitions outside of the prescribed range (i.e., ≥18) during the last set of a particular exercise. Rest periods were 1–2 min between sets and exercises. 

### 2.5. Statistical Analysis

Statistical analyses were performed using SPSS for Windows (SPSS 26.0, Chicago, IL, USA). Mean values were compared using repeated measures analysis of variance (ANOVA) with *a priori* planned contrasts and Bonferroni adjusted statistical significance values. Paired samples *t*-tests were used to determine significant changes in body composition metrics from pre- to post-intervention. Cohen’s d statistic was used for effect sizes (ES), classified as 0.2, 0.5, and 0.8 for small, moderate, and large differences, respectively. The Bland-Altman method was used to determine limits of agreement (LOA) between estimates of body composition [16]. This method identifies the constant error (CE) and 95% confidence interval for the differences (CE ± 1.96 SD) to determine agreement. Proportional bias was assessed by determining the r values (i.e., trends) between the mean (x-axes) and difference (y-axes) of each plot [16]. Linear regression was used to determine the Pearson’s product moment correlation coefficients (*r*) and standard error of the estimate (SEE) for both methods [35]. Total error (TE) or pure error was evaluated with the following formula:

$$\mathrm{TE}=\sqrt{\Sigma }\left(Y-Y^{\prime }\right)/N$$

where Y = observed values, Y′ = predicted values, and N = the number of participants in the sample [40]. Significance was determined as *p* < 0.05, and the strength of each *r* value was described as follows: 0–0.30 small, 0.31–0.49 moderate, 0.50–0.69 large, 0.70–0.89 very large, and 0.90–1.00 near perfect [35]. Standards outlined by Lohman and Heyward were used to qualitatively describe the level of agreement and accuracy observed from each SEE as follows: 2.0 as ideal, 2.5 as excellent, 3.0 as very good, 3.5 as good, 4.0 as fairly good, 4.5 as fair, and 5.0 as poor [31]. All data were expressed as mean ± standard deviation (SD), unless otherwise indicated.

## 3. Results

### 3.1. Participant Characteristics

A total of four boys and six girls (15.8 ± 2.2 yr, 164.0 ± 9.3 cm, 60.0 ± 15.1 kg) were enrolled and completed the study. The girls had higher BMI values at both pre- (25.1 ± 7.4 kg/m^2^) and post-intervention (25.4 ± 7.7 kg/m^2^) assessments than the boys (18.6 ± 1.8 and 19.0 ± 2.2 kg/m^2^, respectively); no significant changes were observed during the intervention. Baseline z-scores were calculated for height (−0.21 ± 0.68), weight (−0.04 ± 1.58), and BMI (−0.1 ± 1.7). Participants had a fasting glucose of 100.8 ± 11.3 mg/dL and a 2 h glucose of 156.2 ± 45.4 mg/dL. Urine-specific gravity values were within accepted ranges at both pre- (1.011 ± 0.011) and post-intervention (1.015 ± 0.007). No significant difference was observed between weight measures obtained from the SECA digital scale, the SFBIA scale, or the MFBIA scale pre-intervention (60.0 ± 16.0, 60.3 ± 16.1, and 60.4 ± 15.9 kg, respectively) or post-intervention (61.3 ± 15.9, 61.4 ± 15.9, and 61.5 ± 15.9 kg, respectively).

### 3.2. Body Composition

**Figure 1:**
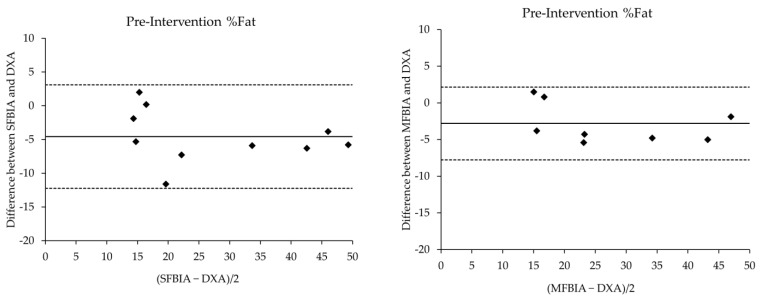

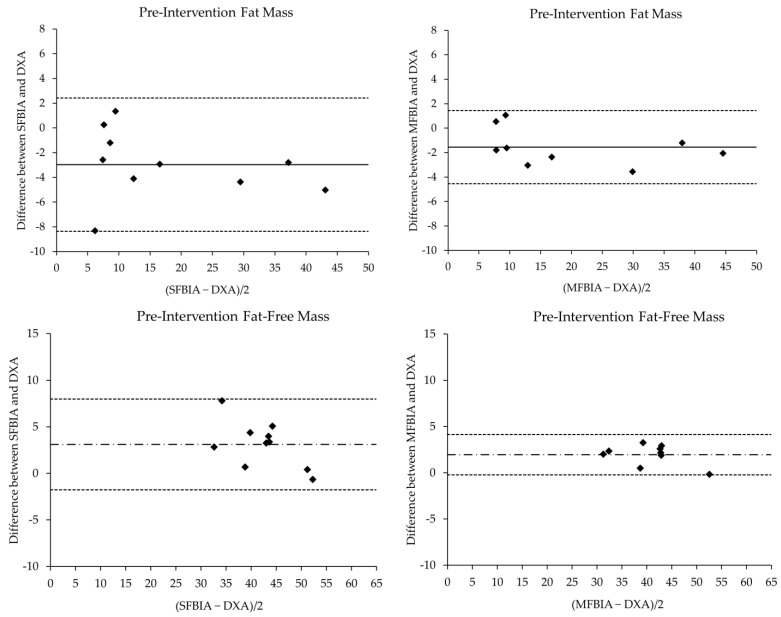
Bland-Altman plots comparing the pre-intervention body composition metrics acquired from single- and multiple-frequency bioelectrical impedance scales and dual energy X-ray absorptiometry. The solid lines represent the mean bias, whereas the outside dashed lines represent the 95% limits of agreement. The black circles represent each participants’ assessment values plotted between absolute difference between methods and mean difference. Fat mass and fat-free mass are expressed in kg. %Fat = percent body fat, DXA = dual energy X-ray absorptiometry, MFBIA = multiple-frequency bioelectrical impedance analysis, SFBIA = single-frequency bioelectrical impedance analysis.

**Figure 2:**
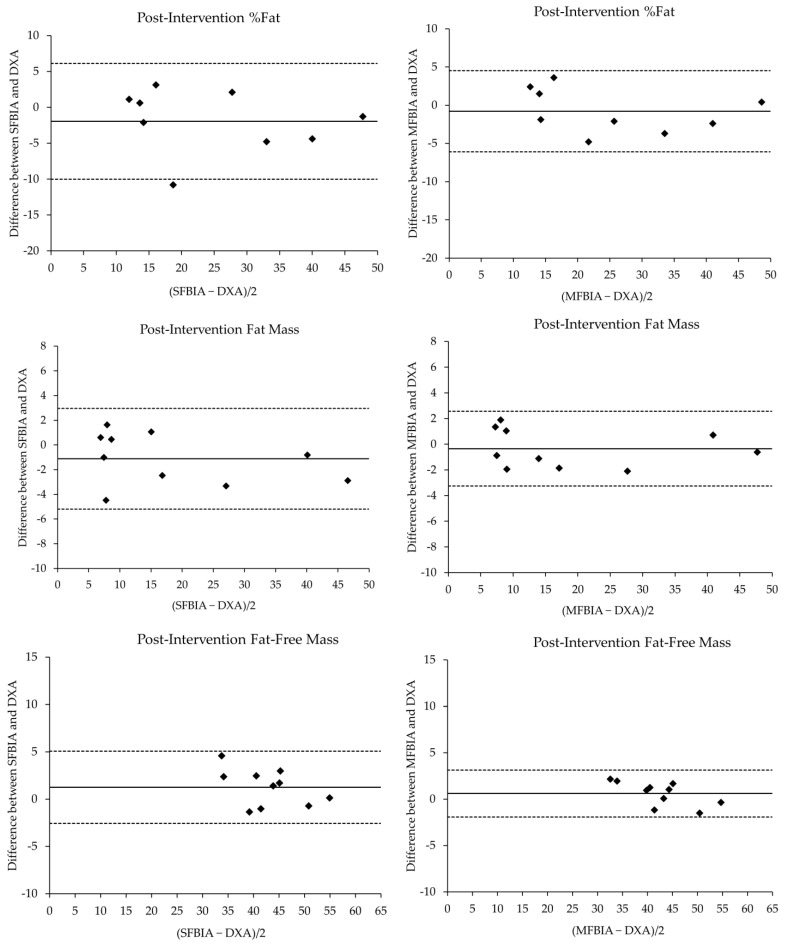
Bland-Altman plots comparing the post-intervention body composition metrics from single- and multiple-frequency bioelectrical impedance scales and dual energy X-ray absorptiometry. The solid lines represent the mean bias, whereas the outside dashed lines represent the 95% limits of agreement. The black circles represent each participants’ assessment values plotted between absolute difference between methods and mean difference. Fat mass and fat free mass are expressed in kg. %Fat = percent body fat, DXA = dual energy X-ray absorptiometry, MFBIA = multiple-frequency bioelectrical impedance analysis, SFBIA = single-frequency bioelectrical impedance analysis.

The comparison statistics for the body composition values of the SFBIA and MFBIA against the DXA are displayed in Table 1. MFBIA was performed in 9 of the 10 participants due to a technical difficulty. Significant, “near perfect” correlations were demonstrated between the DXA and both BIA scales at both time points. The SEE values ranged from “ideal” to “fairly good” in a similar pattern for both pre- and post-intervention comparisons. Bland-Altman plots for pre- and post-intervention agreement between devices are shown in Figure 1 and Figure 2, respectively. At baseline testing, %Fat was underestimated by the SFBIA (mean difference and TE; 4.87% and 1.54) and MFBIA (2.81% and 0.94) compared to DXA. FM was underestimated by SFBIA (2.97 kg and 0.94) and MFBIA (0.44 kg and 0.14) compared to DXA. FFM measures were overestimated by both SFBIA (3.12 kg and 0.99) and MFBIA (0.82 kg and 0.27). At post-intervention testing, %Fat was underestimated by SFBIA (−0.97% and 0.62) and MFBIA (−0.79% and 0.24). A similar trend was observed for FM with SFBIA (−1.12 kg and 0.35) and MFBIA (−0.35 kg and 0.11), with both underestimating relative to DXA. Finally, FFM values by SFBIA (1.25 kg and 0.40) and MFBIA (0.60 kg and 0.19) were overestimated. Table 2 shows changes in body composition metrics before and after the intervention within each method. DXA depicts %Fat and FM reduction with a moderate increase in FFM, while SFBIA displays the opposite trends in all three body composition metrics. MFBIA also demonstrates a similar trend as SFBIA for %Fat and FM; however, it does show a small increase in FFM reflective of DXA.

## 4. Discussion

The current study sought to examine the ability of commercially available BIA scales to quantify body composition among adolescents with CF and to track changes in response to a resistance exercise training intervention. Pre-intervention comparisons demonstrated that both the SFBIA and MFBIA values were significantly different from DXA results, and the effect sizes ranged from small to moderate. Conversely, post-intervention comparisons displayed non-significant differences with small effects on the DXA results and the BIA scales. Near perfect relationships were displayed between the methods with both the SFBIA and MFBIA devices producing values within the limits of agreement for %Fat, FM, and FFM. For tracking body composition changes, DXA results showed mean decreases in %Fat and FM and an increase in FFM. The SFBIA scale demonstrated the exact opposite trend in body composition changes as the criterion method, while the MFBIA scale showed increases in all three metrics from pre- to post-intervention. Both the SFBIA and MFBIA scales selected from the current study underestimate %Fat and FM while overestimating FFM. However, the CE and SEE values were consistently smaller with the MFBIA device for all outcomes than the SFBIA when compared to DXA results. The Bland-Altman analysis reflects these results where the 95% limits of agreement are larger for SFBIA-derived metrics compared to the MFBIA. Though MFBIA appears to be superior to SFBIA, body composition assessment results from portable BIA scales should be interpreted with caution.

With the number of portable BIA available to the public consistently growing over the last decade, a greater need has arisen to investigate their ability to accurately measure body composition, specifically in children and adolescents. Wang and Hui [38] examined the validity of four BIA consumer devices to predict %Fat in 255 healthy Chinese children and adolescents aged 9–19 years. Lee et al. [37] looked at the validity of two portable BIA devices in 150 healthy Taiwanese children aged 6–12. Both studies compared single-frequency and multi-frequency devices to DXA and found the multi-frequency devices to be superior to single-frequency BIA. However, in both studies, the multi-frequency BIA device underestimated %Fat and overestimated FFM, similar to the results of the current study [37,38]. Wang and Hui [38], as well as Lee et al. [37], reached the conclusion that BIA measurement could be useful for health practitioners in assessing body composition in children and adolescents, but results should be reviewed carefully, and that additional validation studies in specific subpopulations are required.

The findings of the current study fall in line with previous research examining the utility of BIA assessments in patients with CF. King et al. [34] compared body composition measures among 76 adults with CF (29.9 ± 7.9 yr) using DXA, SFBIA with two prediction equations (Lukaski [41] and Segal [42]), and skinfold thickness. Both SFBIA equations produced values that were strongly correlated with FFM results from DXA (Lukaski *r* = 0.95; Segal *r* = 0.94). However, the BIA equation of Lukaski significantly underestimated FFM in men compared to DXA (51.5 ± 7.8 vs. 54.8 ± 7.3 kg), while the BIA equation of Segal overestimated FFM in women compared to DXA (44.1 ± 5.9 vs. 41.2 ± 3.9 kg). With large differences and wide LOA when comparing on an individual basis, King et al. concluded FFM bias from BIA was inconsistent, especially for women, and could result in misclassification of nutritional status [34]. Ziai et al. [35] investigated the agreement between SFBIA and DXA in 34 adults with CF (30 ± 9 yr). The results displayed strong correlations for FFM (0.915), FM (0.914), and %Fat (0.833); however, BIA overestimated FFM in individuals with <40 kg of FFM and underestimated FM and %Fat in people with <20 kg of FM. Ziai et al. concluded that if BIA error remained constant over time, then the impact for clinicians assessing body composition changes may not be major but could be significant when phenotyping patients [35]. Finally, Charatsi et al. [33] examined 54 CF patients using DXA and MFBIA with an equation specific for adolescents with CF. FFM from BIA and DXA were significantly correlated (0.995), with a small mean difference (−4.87e−15 kg) and tight LOA (−2.25 to 2.25). Similarly, FM was highly correlated (0.96), with a small mean difference (0.6 kg) and tight LOA (−1.82 to 3.02), concluding that BIA is a valid measure of body composition in CF patients [33].

In the present study, the agreement between BIA and DXA measurements increased from pre- to post-intervention. Lands et al. [43] examined the agreement of lean body mass measurements assessed using skinfold measures, SFBIA, and DXA in 12 young CF patients (11.2 ± 2.7 yr) twice over a 6 month period. Baseline values from DXA (31.2 ± 9.8 kg) and BIA (30.0 ± 9.0 kg) were significantly different from one another, with LOA ranging from 1.88 to −4.28 kg. At six months, no significant differences were observed between DXA (33.9 ± 10.5 kg) and BIA (32.8 ± 9.4 kg), although LOA was wider at 5.53 to −7.79 kg. Additionally, no significant correlations were observed among the three techniques [43]. In the current study, the same pre-testing guidelines and BIA testing procedures were followed pre- and post-intervention, so it is unclear why agreement differed between time points. Future research should examine the reliability of portable BIA devices, as well as the effects of BIA validity following an exercise intervention designed to induce body composition changes.

It is suggested that the accuracy of BIA measurements of body composition is dependent on BMI and sex. In the current study, discrepancies were found between boys and girls, specifically with FFM measures; however, due to the small sample size and unequal groups, it is difficult to tell how much sex differences play a role. Boneva and Boyanov [44] found that SFBIA underestimated FM and %Fat and overestimated FFM in lean individuals when compared to DXA. However, this trend reversed at a BMI >35 kg/m^2^. Moreover, strong correlations were observed between the two methods but decreased as BMI increased (*r* = 0.82–0.95). Boneva and Boyanov concluded that BIA was an accurate method, but caution should be taken when assessing obese women (BMI >35 kg/m^2^) [44]. Castro et al. [45] found that the InBody 720 compared to DXA demonstrated significant correlations in 64 children and adolescents (12.2 ± 2.1 yr) for FFM and lean body mass (Rho = 0.901–0.992) with a trend to overestimate in both sexes. However, clinically unacceptable correlations were obtained for %Fat, FM, and bone mineral content (Rho = 0.744–0.862) with a trend of underestimating in both sexes. These errors increased as body composition metrics increased via DXA [45]. Larsen et al. [46] investigated the validity of the InBody 270 against DXA in children (10.7 ± 0.5 yr). For both the boys and girls, lean body mass was underestimated by the InBody (16.7 ± 2.7 and 16.3 ± 2.7 kg, respectively) compared to DXA (27.7 ± 3.9 and 27.3 ± 4.3 kg, respectively). In the boys, FM values were underestimated between DXA (10.8 ± 5.1 kg) and InBody (8.3 ± 4.6 kg) but overestimated in the girls (12.1 ± 4.4 and 24.5 ± 5.5 kg, respectively) [46].

An advantage of BIA over DXA is the ability to estimate total body water through its measure of impedance. In contrast, DXA is unable to assess body water and therefore assumes a constant level of hydration, which introduces error because total body water varies among individuals [22]. The normal daily fluctuations in hydration status among humans and the small difference in urine-specific gravity between pre- and post-intervention assessments may have contributed to the differences observed between DXA and BIA. Additionally, the MFBIA scale demonstrated superior agreement, stronger correlations, and small effect sizes in comparison to the SFBIA scale. These results may be indicative of the MFBIA device’s ability to use multiple frequencies to differentiate intracellular from extracellular fluid, providing more accurate estimates of body composition [37]. The superiority of MFBIA devices to SFBIA has been demonstrated in previous research studies in both children and adults, primarily attributed to MFBIA’s ability to measure impedance, reactance, and resistance across a greater range of frequencies allowing for a more comprehensive assessment of body composition relative to DXA [29,31,37,47].

Limitations of the present study include the small sample size, which limits the statistical power and ability to stratify data based on sex or BMI. Enrolling patients with CF for an intervention study is difficult, especially when the eligibility criteria are stringent, as in our study. However, this was a pilot study meant to collect preliminary data for future research in a larger sample. Moreover, even with the small number of participants, the results fall in line with previous literature. Another limitation was a lack of measurements for reliability testing. Future studies should look to repeat measurements within the same week to assess the reliability and between-day variations while participants are weight stable. The selected BIA scales can provide estimates of multiple variables, including muscle mass, total body water, and bone mass. Due to the age restrictions by the BIA scales, these measurements could not be obtained for comparison to DXA. Finally, a limited number of BIA devices were utilized in this study. With the wide variety of portable BIA scales commercially available, all of which utilize manufacturer-specific estimation equations, comparisons of a greater number of devices are recommended. Because the body composition prediction equations utilized in commercially available BIA scales are unique to the specific device and manufacturer, the differences observed among SFBIA and MFBIA in the current study may be a side-effect of the scales selected and not BIA as a whole.

## 5. Conclusions

Bioelectrical impedance devices provide a versatile method for accessing body composition and tracking changes on a frequent basis without risk, high cost, or significant expertise to operate. In the current study, both the SFBIA and MFBIA offered a good agreement, near perfect correlations, and small-moderate effect sizes at two different time points; however, significant differences from DXA were detected at baseline. Furthermore, wide limits of agreement were demonstrated alongside inconsistent variability in CE. Considering all comparison statistics analyzed, MFBIA performed the more accurate measurements in contrast to SFBIA with DXA as the criterion. These results may be indicative of MFBIA’s ability to use multiple frequencies to differentiate intracellular from extracellular fluid, providing more accurate estimates of body composition [37]. We conclude that BIA appears to be an acceptable method for assessing body composition, with MFBIA being superior to SFBIA. Because DXA scans are not regularly implemented in clinical settings to assess body composition in individuals with CF for a number of reasons, including time constraints for clinicians, the expense to utilize, and technical expertise required, MFBIA may be used in a home-based setting, for the personal assessment of body composition and tracking of changes during or following an exercise training program. In this way, patients with CF can gain insight into body composition changes. However, commercially available BIA scales may not be suitable as a *replacement* method for DXA when clinically evaluating patients with CF. 

## Figures and Tables

**Table 1:** Comparison of body composition metrics between DXA and BIA devices pre- and post-intervention.

Pre-Intervention	N	Mean ± SD	*p*-Value	Effect Size		*r*	SEE	CE ± 1.96SD	95% LOA	
									Lower	Upper
**%Fat Total**										
DXA	9	31.29 ± 14.66	---	---	---	---	---	---	---	---
SFBIA	9	26.42 ± 13.81 *	0.01	0.34	Moderate	0.97 *	3.81	−4.57 ± 1.14	−12.32	3.18
MFBIA	9	28.48 ± 13.93 *	0.01	0.20	Moderate	0.99 *	2.52	−2.81 ± 0.74	−7.80	2.18
**FM (kg)**										
DXA	9	19.27 ± 14.08	---	---	---	---	---	---	---	---
SFBIA	9	16.30 ± 13.39 *	0.01	0.21	Moderate	0.98 *	2.74	−2.97 ± 0.80	−8.38	2.44
MFBIA	9	18.83 ± 13.90 *	0.02	0.03	Small	1.00 *	1.49	−1.56 ± 0.44	−4.54	1.42
**FFM (kg)**										
DXA	9	40.77 ± 7.19	---	---	---	---	---	---	---	---
SFBIA	9	43.89 ± 5.70 *	<0.01	0.48	Moderate	0.95 *	1.86	3.12 ± 0.73	−1.86	8.10
MFBIA	9	41.59 ± 6.13 *	<0.01	0.12	Small	0.99 *	1.03	1.96 ± 0.35	−0.43	4.34
**Post-Intervention**										
**%Fat Total**										
DXA	10	28.35 ± 15.34	---	---	---	---	---	---	---	---
SFBIA	10	26.38 ± 14.42	0.16	0.13	Small	0.96 *	4.08	−1.97 ± 1.14	−9.70	5.76
MFBIA	10	27.56 ± 14.56	0.38	0.05	Small	0.99 *	2.68	−0.79 ± 0.75	−5.85	4.27
**FM (kg)**										
DXA	10	18.99 ± 15.02	---	---	---	---	---	---	---	---
SFBIA	10	17.87 ± 14.28	0.12	0.08	Small	0.99 *	2.01	−1.12 ± 0.58	−5.04	2.80
MFBIA	10	18.64 ± 14.80	0.48	0.02	Small	1.00 *	1.54	−0.35 ± 0.41	−3.12	2.42
**FFM (kg)**										
DXA	10	42.30 ± 7.18	---	---	---	---	---	---	---	---
SFBIA	10	43.55 ± 6.29	0.07	0.19	Small	0.97 *	1.71	1.26 ± 0.54	−2.44	4.95
MFBIA	10	42.90 ± 6.31	0.17	0.09	Small	0.99 *	0.95	0.60 ± 0.36	−1.82	3.03

%Fat = percent body fat, CE = constant error; DXA = dual energy X-ray absorptiometry, FFM = fat free mass, FM = fat mass, LOA = limits of agreement, MFBIA = multiple-frequency bioelectrical impedance analysis, SD = standard deviation, SEM = standard error of the mean, SFBIA = single-frequency bioelectrical impedance analysis; * significant alpha level at *p* < 0.05.

**Table 2:** Pre- and post-intervention differences in body composition metrics within each assessment method.

Mean ± SD				Pre-Post Differences							
	N	Pre	Post	Mean Difference	Effect Size		SD	SEM	95% CI Diff		*p*-Value
									Lower	Upper	
**DXA**											
%Fat	10	29.69 ± 14.72	28.35 ± 15.34	−1.34 *	0.09	Small	1.59	0.50	0.20	2.48	0.03
FM (kg)	10	19.27 ± 14.08	18.99 ± 15.02	−0.29	0.02	Small	1.68	0.53	−0.92	1.49	0.60
FFM (kg)	10	40.77 ± 7.19	42.30 ± 7.18	1.53 *	0.21	Moderate	1.43	0.45	−2.55	−0.50	0.01
**SFBIA**											
%Fat	10	25.12 ± 13.66	26.38 ± 14.42	1.26	0.09	Small	3.62	1.15	−3.85	1.33	0.30
FM (kg)	10	16.30 ± 13.39	17.87 ± 14.28	1.57	0.11	Small	2.58	0.81	−3.41	0.28	0.09
FFM (kg)	10	43.89 ± 5.70	43.55 ± 6.29	−0.34	0.05	Small	2.76	0.87	−1.63	2.32	0.71
**MFBIA**											
%Fat	9	28.48 ± 13.93	29.09 ± 14.57	0.61	0.04	Small	1.96	0.65	−2.12	0.90	0.38
FM (kg)	9	18.83 ± 13.90	19.83 ± 15.19	1.00	0.07	Small	1.91	0.64	−2.47	0.48	0.16
FFM (kg)	9	41.59 ± 6.13	42.14 ± 6.20	0.55	0.09	Small	1.76	0.59	−1.91	0.80	0.37

%Fat = percent body fat, DXA = dual energy X-ray absorptiometry, FFM = fat free mass, FM = fat mass, M = mean, MFBIA = multiple-frequency bioelectrical impedance analysis, SD = standard deviation, SEM = standard error of the mean, SFBIA = single-frequency bioelectrical impedance analysis; * significant alpha level at *p* < 0.05.

## Data Availability

This study did not report any public data.
